# Intestinal current measurement versus nasal potential difference measurements for diagnosis of cystic fibrosis: a case–control study

**DOI:** 10.1186/1471-2466-14-156

**Published:** 2014-10-04

**Authors:** Azadeh Bagheri-Hanson, Sebastian Nedwed, Claudia Rueckes-Nilges, Lutz Naehrlich

**Affiliations:** Department of Pediatrics, Justus-Liebig-University Giessen, Feulgenstrasse 12, 35385 Giessen, Germany

**Keywords:** (3–10): Cystic fibrosis, Nasal potential difference, Intestinal current measurement, Sweat chloride, Sweat test, Diagnosis, Smoking

## Abstract

**Background:**

Nasal potential difference (NPD) and intestinal current measurement (ICM) are functional *CFTR* tests that are used as adjunctive diagnostic tools for cystic fibrosis (CF). Smoking has a systemic negative impact on *CFTR* function. A diagnostic comparison between NPD and ICM and the impact of smoking on both *CFTR* tests has not been done.

**Methods:**

The sweat chloride test, NPD, and ICM were performed in 18 patients with CF (sweat chloride >60 mmol/l), including 6 pancreatic sufficient (PS) patients, and 13 healthy controls, including 8 smokers. The NPD *CFTR* response to Cl-free and isoproterenol perfusion (Δ0Cl^-^ + Iso) was compared to the ICM *CFTR* response to forskolin/IBMX, carbachol, and histamine (ΔI_sc, forskolin/IBMX+ carbachol+histamine_).

**Results:**

The mean NPD *CFTR* response and ICM *CFTR* response between patients with CF and healthy controls was significantly different (p <0.001), but not between patients with CF who were PS and those who were pancreatic insufficient (PI). Smokers have a decreased CFTR response measured by NPD (p = 0.049). For ICM there is a trend towards decreased CFTR response (NS). Three healthy control smokers had NPD responses within the CF-range. In contrast to NPD, there was no overlap of the ICM response between patients with CF and controls.

**Conclusions:**

ICM is superior to NPD in distinguishing between patients with CF who have a sweat chloride > 60 mmol/l and healthy controls, including smokers. Neither NPD nor ICM differentiated between patients with CF who were PS from those who were PI. Smoking has a negative impact on *CFTR* function in healthy controls measured by NPD and challenges the diagnostic interpretation of NPD, but not ICM.

**Electronic supplementary material:**

The online version of this article (doi:10.1186/1471-2466-14-156) contains supplementary material, which is available to authorized users.

## Background

Cystic fibrosis (CF) is diagnosed based on a defined clinical phenotype and confirmation of *cystic fibrosis transmembrane regulator* (*CFTR*) dysfunction, commonly demonstrated by a sweat chloride value of ≥ 60 mmol/l and/or detection of two CF-causing mutations [1, 2]. A small but increasing number of patients present with clinical symptoms characteristic of CF, an intermediate (30–60 mmol/l) or negative (≤29 mml/l) sweat test, and less than two CF-causing mutations [3–6]. For these query CF patients, a specific and sensitive *CFTR* functional test to exclude or confirm a *CFTR* functional defect characteristic of CF is needed [1, 6]. *CFTR* modulating and correcting drugs have improved *CFTR*-function in cell cultures [7]. To test their effect in CF-patients, especially those with rare mutations, *CFTR*-functional tests with low variability and high reproducibility are needed [7].

In addition to sweat testing, two additional *CFTR* functional tests have been developed over the past 30 years; nasal potential difference (NPD) measurement [8] and intestinal current measurement (ICM) [9]. *CFTR* function is measured *in vivo* in the respiratory epithelium by NPD and *ex vivo* in superficial rectal biopsies by ICM. International standard operating procedures (SOPs) have been established for both tests [8, 10]. Both tests block epithelial sodium channels by amiloride and stimulate cAMP-mediated CFTR-mediated chloride transport in chloride-free solution and isoproterenol (NPD) or forskolin and IBMX (ICM). In addition, cholinergic chloride transport is tested by carbachol with ICM. The change after chloride-free and isoproterenol perfusion (Δ0Cl^-^ + Iso) with NPD [8] and the sum of the responses after carbachol, forskolin/IBMX ([11]) plus histamine (ΔI_sc, forskolin/IBMX+ carbachol+histamine_) with ICM [12] has been proposed to be the best parameter of *CFTR* function. Both techniques can discriminate CF patients from healthy controls (NPD [13, 14]; ICM [11, 12, 15, 16]), but comparative clinical trials are lacking. Smoking has a systemic negative impact on CFTR-function [17, 18], but the impact on the diagnostic aspects of NPD and ICM have not been investigated.

We performed NPD and ICM measurement in CF-patients and healthy controls to determine (1) the ability of these measurements to differentiate CF patients from healthy controls, and (2) the influence of smoking on CFTR function in healthy controls.

## Methods

Between October 2012 and February 2013, 18 patients with CF and 13 healthy controls were recruited at the Justus-Liebig-University, Giessen, Germany. For this study, the diagnosis of CF was based on at least one clinical manifestation of CF, sweat chloride ≥60 mmol/l and the presence of two CF-causing mutations [1]. Pancreatic sufficiency (PS) was defined as fecal elastase >100 μg/g stool. Healthy controls had no clinical manifestation of CF and a sweat chloride value <60 mmol/l (Non-CF). Smoking was defined as any active or passive exposure to tobacco smoke. Exclusion criteria were participation in another medical clinical trial during the past 30 days, acute respiratory symptoms, intake of ivacaftor, known hemorrhoids, or bleeding diathesis. The ethics committee of the Justus-Liebig-Universität Giessen approved the protocol (AZ109/12). The study was performed in accordance with the declaration of Helsinki. Written informed consent was obtained from each participant aged 18 years and older. For participants younger than 18 years of age, written informed consent was obtained from each participant’s parents or legal guardian, and age-appropriate consent was obtained from each participant. The sweat test, NPD, and ICM were performed on the same day for each subject.

The sweat test was performed according to Clinical and Laboratory Standards Institute guidelines [19]. For sweat stimulation and collection, the Macroduct® system (Wescor, Inc., Logan, USA) was used. Chloride was measured by chloride titration. A sweat chloride level ≥60 mmol/l was interpreted as within the CF range, 30–60 mmol/l as equivocal, and ≤29 mmol as normal [1].

NPD was performed by one operator who was accredited by the Cystic Fibrosis Foundations’ (CFF) Therapeutic Developments Network (TDN), and followed the CFF TDN SOP (version: January 2009) [8]. We used terbutaline as a substitute for isoproterenol in accordance with the SOP. The chloride-free and isoproterenol response (Δ0Cl^-^ + Iso) (NPD *CFTR* response) and the Wilschanksi score (defined as e^(response to chloride-free and isoproterenol/response to amiloride)^) [20] representing the *CFTR* response were calculated as the average or best result from both nostrils. The Δ0Cl^-^ + Iso was interpreted as normal when < -12 mV, as in the CF range when > -7.7 mV, and as equivocal for results between -12 and -7.7 mV [6]. The Wilschanski score was interpreted as normal (<0.65), in the CF-range (>0.70), or equivocal (0.65–0.70) [20]. If the mean Δ0Cl^-^ + Iso was > -7.7 mV in healthy controls, the NPD was repeated on a different day. Only the measurement with the highest Δ0Cl^-^ + Iso was reported. If both measurements confirmed a Δ0Cl^-^ + Iso in the CF range, *CFTR* genotyping (sequencing and multiplex ligation-dependent probe amplification) was offered as part of the participant’s clinical care and reported as part of the baseline data. Genotyping of all healthy controls was not ethically approved.

The ICM followed the European Cystic Fibrosis Society-Therapeutic Development Network (ECFS-TDN) SOP (V2.7; Oct 26, 2011), which is based on the Rotterdam protocol. The tissues sliders (P2407C [1.5 mm diameter aperture slider; area 0,018 cm^2^] or P2407B [1.2 mm diameter aperture slider; area 0.011 cm^2^]; Physiologic Instruments, San Diego, USA) were mounted without tissue in the chambers (4-chamber system [EM-LVSYS-4; Physiologic Instrument, San Diego, USA]), which were filled on both sides with 2 ml Meyler buffer solution (10 mM Hepes; 0.3 mM Na_2_HPO_4_; 0.4 mM NaH_2_PO_4_; 1.0 mM MgCl_2_; 1.3 mM CaCl_2_; 4.7 mM KCl; 128 mM NaCl; 20.2 mM NaHCO_3_; 10 mM D-Glucose; 0.01 mM indomethacin; pH 7.4; osmolarity 300 mOsm). PowerLab (4/30; ADInstruments Ltd., Dunedin, New Zealand) and Labchart® software (release 7.2; ADInstruments Ltd., Dunedin, New Zealand) were used for data acquisition. A stable open Potential Difference (PD) was ensured and an input offset to 0 mV was performed. Fluid resistance compensation was performed by applying short current pulses (15 μA) by the VCC MC4S Multi-Channel Voltage Current Clamp (Physiologic Instrument, San Diego, USA) and adjusting the fluid resistance.

For ICM, at least 4 superficial rectal biopsies were obtained by suction biopsies (aspiration biopsy instrument according to Wilital (UE7605); ulrich GmbH, Ulm, Germany) without prior bowel preparation. Biopsies were immediately stored in ice-cold buffer solution (Dulbecco’s phosphate buffered saline and indomethacin, final concentration 10 μM) and mounted on adequate tissue sliders. After mounting the sliders in the heated and slightly sparged (95% O_2_/5% CO_2_) 4-chamber system, each basal resistance was measured by applying short current pulses (15 μA) and registering the corresponding change in V_t_ (typical range 15–30 Ohm × cm^2^) with the VCC MC4S Multi-Channel Voltage Current Clamp (Physiologic Instrument, San Diego, USA). After that the voltage was clamped at 0 mV and the raw short circuit current (_r_I_sc_) was recorded from then on. Due to different sliders with different areas (P2407C [1.5 mm diameter aperture slider; area 0.018 cm^2^] or P2407B [1.2 mm diameter aperture slider; area 0.011 cm^2^]); Physiologic Instruments, San Diego, USA), the raw _r_I_sc_ was converted to I_sc_ (μA/cm^2^). After applying 100 μM carbachol (which stimulates cholinergic Cl^-^ secretion by opening basolateral K^+^ channels) to the serosal compartment, an I_sc_ response was evoked for quality control of the biopsy. After a 40-min equilibration in Meyler buffer, the basal I_sc_ was noted and 2 μl amiloride (to block amiloride-sensitive sodium channels) was added to the mucosal compartment. After 5 min or when the I_sc_ was stable, 10 μM forskolin and 100 μM IBMX (to stimulate cAMP-dependent CFTR-Cl^-^ transport) were added to the mucosal and serosal compartments (ΔI_sc, forskolin/IBMX_). After a minimum of 10 min, 10 μM genisteine (CFTR-potentiator) was added to both compartments. After a minimum of 5 min, 100 μM carbachol was added to the serosal compartment (ΔI_sc, carbachol_). After a minimum of 10 min, 200 μM 4,4′-Diisothiocyano-2,2′-stilbenedisulfonic acid (DIDS) (blocking non-CFTR-Cl^-^ channels) was added to the mucosal compartment. After 10 min, 500 μM histamine (to stimulate Ca^2+^ and proteinkinase C-mediated CFTR Cl^-^ secretion) was added to the serosal compartment (ΔI_sc, histamine_). In the open circuit, the final transepithelial voltage and final resistance were measured by applying short current pulses as in the beginning. Preliminary data suggested that the average ΔI_sc, forskolin/IBMX+ carbachol+histamine_ is the best diagnostic ICM parameter for chloride secretory response (ICM CFTR response), but reference ranges have not been established [10].

Data are presented as the mean ± standard deviation (SD) (normally distributed variables), respective the median values, and 25th and 75th percentiles (non-normally distributed variables). Group comparisons were performed using the Student’s t test or Mann–Whitney U test for normally or non-normally distributed variables, respectively. Statistical significance was defined as *p* < 0.05. All analyses were performed with IBM SPSS Statistics 21 (release 21; IBM, Armonk, USA).

## Results

**Figure 1:**
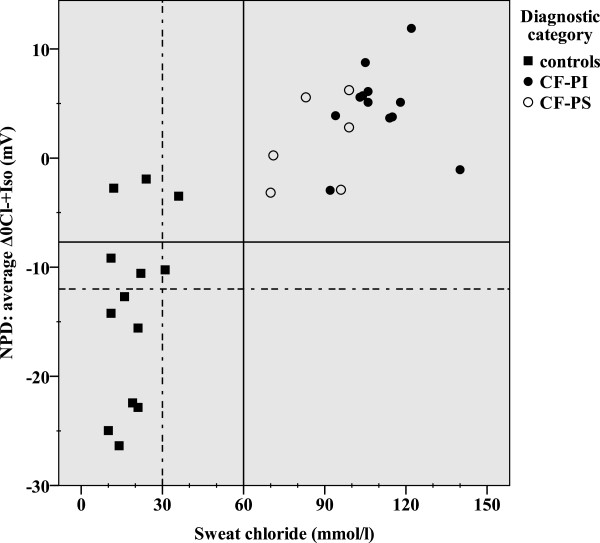
**Correlation of average Δ0Cl** ^**-**^ **+ Iso (NPD) and sweat chloride.** The normal range is indicated for values below and left of the dotted lines, and the intermediate range is shown between the solid and dotted lines.

**Figure 2:**
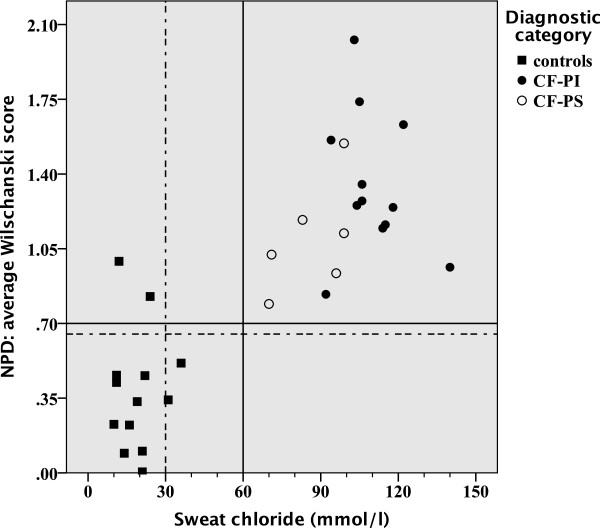
**Correlation of the average Wilschanski score (NPD) and sweat chloride.** The normal range is shown below and left of the dotted lines, and the intermediate range is shown between the solid and dotted lines.

**Figure 3:**
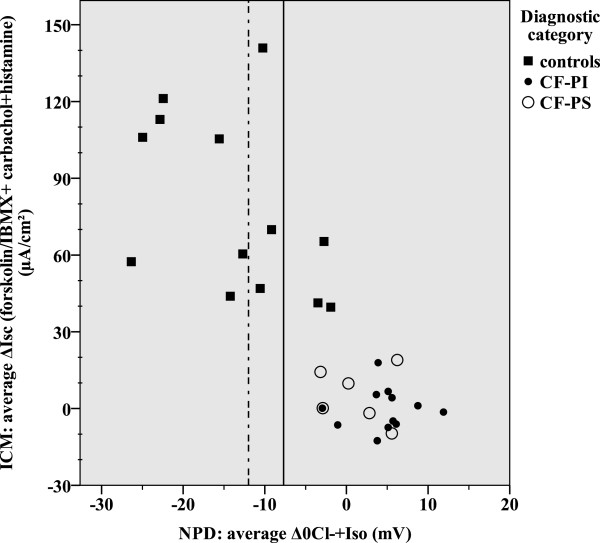
**Correlation between the average ΔIsc (forskolin/IBMX + carbachol + histamine) (ICM) and average Δ0Cl** ^**-**^ **+ Iso (NPD).** The normal range is shown left of the dotted line. The intermediate range is shown between the solid and dotted lines. A higher ΔIsc (forskolin/IBMX + carbachol + histamine) represents a better CFTR response.

**Figure 4:**
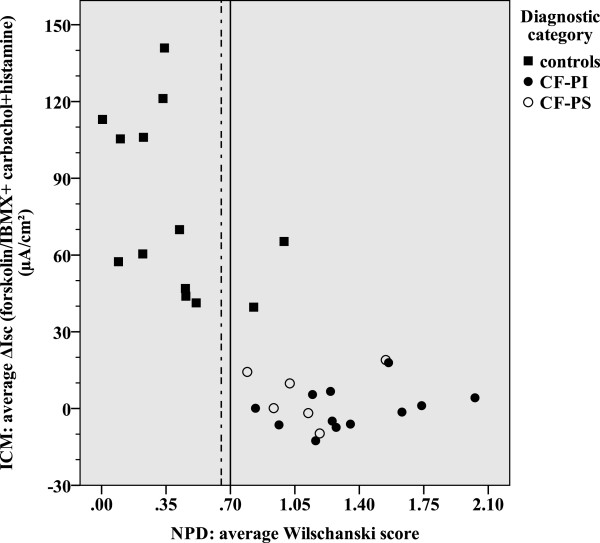
**Correlation between the average ΔIsc (forskolin/IBMX + carbachol + histamine) (ICM) and average Wilschanski score (NPD).** The normal range is shown left of the dotted line. The intermediate range is shown between the solid and dotted lines. A higher ΔIsc (forskolin/IBMX + carbachol + histamine) represents a better CFTR-response.

Our study included 18 patients with CF and 13 healthy controls with a median age of 20.5 and 25.0 years, respectively (NS) (Table 1). As consequence of our inclusion criteria, sweat chloride values distinguish patients with CF from healthy controls (p < 0.001). Although the mean sweat chloride differed between CF-PS and CF-PI patients (p = 0.003), an individual overlap occurs (Table 1, Figure 1). The mean NPD *CFTR* response significantly discriminated between CF patients and healthy controls (p <0.001), but not between patients with CF who were PS versus PI (Table 1). Four healthy controls had an average Δ0Cl^-^ + Iso of > -7.7 mV. Three out of four controls had a repeatable average NPD CFTR response in the CF-range for Δ0Cl^-^ + Iso > -7.7 mV (23% of all healthy controls) (Figure 1), and two additional controls when using the Wilschanski score (15% of all healthy controls) (Figure 2). All these healthy controls were smokers. CFTR genotyping was offered to these three healthy controls as part of clinical routine and none had two CF-causing mutations (Table 1). For ICM a median of 6 (5–7) rectal biopsies were sampled per patient without severe adverse events. The mean ICM CFTR response was significantly different between CF patients and healthy controls (p <0.001), but not between patients with CF who were PS versus those who were PI (Table 1). We could not detect any age-dependency of the response to Isoproterenol/Forskolin. In contrast to NPD, there was no overlap between CF-patients and controls (Figures 3 and 4). Using the best instead of the average NPD, the *CFTR* response overlap did not change (Additional file 1). Using the best instead of the average ICM, the *CFTR* response resulted in one overlap (Additional file 2).

**Table 1:** **Characteristics and CFTR response of pancreatic insufficient (CF-PI) and pancreatic sufficient (CF-PS) patients with CF and controls**

	CF-PI (n = 12)	CF-PS (n = 6)	CF-all (n = 18)	Controls (n = 13)
**Age, years**	24.0 ± 6.1	23.3 ± 11.8	22.8 ± 8.0	30.6 ± 10.4
	22.0 (19.0 – 26.0)	16.0 (14.5 – 30.5)	20.5 (18.3 – 25.3)	25.0 (23.5 – 35.5)
**Gender, females:males**	3:9	5:1	8:10	7:6
**Body mass index Z-score**	-1.18 ± 0.80	-0.62 ± 1,41	-0.99 ± 1.03*	0.00 ± 0.65*
	-1.05 (-2.40 – 0.00)	1.41 (-0.20 – 0.70)	-0.90 (-2.60 – 0.70)	0.00 (-1.10 - 1.30)
**Sweat chloride (mmol/l)**	110 ± 13^**^	86 ± 14^**^	102 ± 17*	19 ± 8*
	106 (92 – 140)	90 (70 – 99)	104 (70 – 140)	19 (10 – 36)
**NPD** ***CFTR*** **response average Δ0Cl** ^**-**^ **+ Iso (mV)**	4.6 ± 3.9	1.5 ± 4.1	3.6 ± 4.1*	-13.6 ± 8.5*
	5.1 (-3.0 -11.9)	1.5 (-3.2 – 6.23)	4.5 (-3.2 – 11.9)	-12.7 (-26.4 - -1.92)
**ICM** ***CFTR*** **response average ΔIsc (μA/cm** ^**2)**^ **) (forskolin/IBMX + carbachol + histamine)**	-0.3 ± 8.1	5.3 ± 10.9	1.6 ± 9.2*	77.8 ± 34.8*
	-0.6 (-12.6 – 17.9)	5.0 (-9.7 – 19.0)	0.1 (-12.6 – 19.0)	65.3 (39.6 -140.9)
**Genotyping**	F508/F508 (6×)	F508/R347P (2×)		148 T/R117H-7 T
	F508/G551D (2×)	F508/3849 + 10 kb C- > T (2×)		F508/--
	F508/G542X	F508/R334W		--/--
	F508/N1303K	F508/?		ND/ND (11)
	F508/1248 + 1G-A			
	F508/dele 14a,15,16,17a,17b			

Data are shown as ratios or the mean ± standard deviation incl. median (min – max). *p <0.001 (CF-all versus controls); **p = 0.003 (CF-PI versus CF-PS).

**Figure 5:**
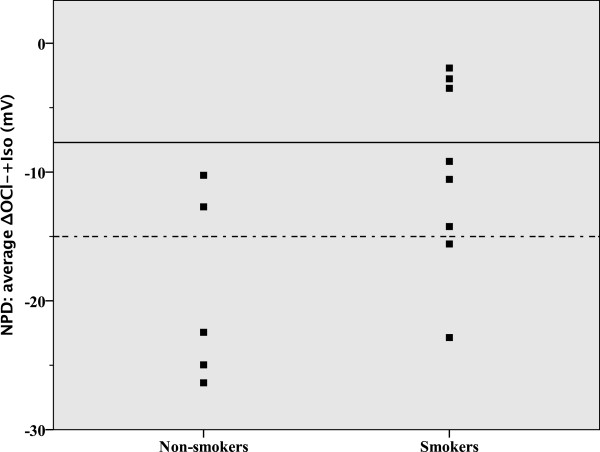
**Average Δ0Cl** ^**-**^ **+ Iso (NPD) in healthy controls according to smoking status.** The normal range is shown below the dotted line and the intermediate range is shown between the solid and dotted lines.

**Figure 6:**
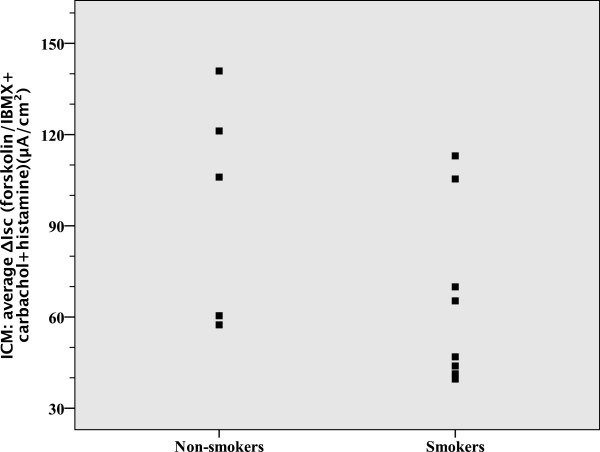
**Average ΔIsc (forskolin/IBMX + carbachol + histamine) (ICM) in healthy controls according to smoking status.** A higher ΔIsc (forskolin/IBMX + carbachol + histamine) represents a better CFTR response.

In healthy controls, smoking had no influence on sweat chloride (NS), but decreased *CFTR* function as measured by NPD (p = 0.049) (Table 2 and Figure 5), and resulted in intermediate or even abnormal NPD results, but not ICM or sweat chloride results. There is a trend in ICM measurements indicative that smoking might not only affect CFTR in the respiratory tract, but also in the intestine (Table 2 and Figure 6).

**Table 2:** **Influence of smoking (active and/or passive) in healthy controls on NPD and ICM** ***CFTR*** **responses**

	Nonsmokers	Smokers	***p-value***
	n = 5	n = 8	
Sweat chloride (mmol/L)	18 ± 8	20 ± 9	NS
NPD *CFTR* response	-19.3 ± 7.4	-10.1 ± 7.3	0.049
Average Δ0Cl^-^ + Iso (mV)			
ICM *CFTR* response	97.2 ± 37.1	65.7 ± 29.1	NS
Average ΔIsc (μA/cm^2^)			
(forskolin/IBMX + carbachol + histamine)			

All values are shown as the mean ± standard deviation.

## Discussion

ICM is superior to NPD for distinguishing between CF patients with a sweat chloride >60 mmol/l and healthy controls, including smokers. Neither NPD nor ICM differentiated patients with CF who were PS from those who were PI. Smoking has a negative impact on *CFTR* function in healthy controls measured by NPD, and challenges the diagnostic interpretation of NPD. There is a trend in ICM indicative that smoking might not only affect CFTR in the respiratory tract, but also in the intestine, which has no impact on diagnostic interpretation.

NPD has been used as a diagnostic test for CF since the late 1980s [14, 21, 22]. Studies have shown 94.8–100% sensitivity and 96.5–100% specificity of Δ0Cl^-^ + Iso for separating PI patients with CF from healthy controls [13, 14, 23]. Experience with a broader spectrum of patients with CF [24, 25] and equivocal patients (sweat chloride <60 mmol/l and less than two CF-causing mutations) [20] described a clinically relevant overlap for Δ0Cl^-^ + Iso. Even in F508del homozygous patients, a residual *CFTR* NPD response with [26] or without [27, 28] an observed clinical difference has been described. Some centers introduced an intermediate category for Δ0Cl^-^ + Iso [6], interpret the highest NPD CFTR response [29], or use a composite score that includes sodium and chloride conductance [20, 30]. Irrespective of the diagnostic criteria, our result showed a clinically relevant overlap between patients with CF and healthy controls. A normal average NPD *CFTR* response excludes CF, but an abnormal average NPD CFTR response could occur in healthy controls, especially in smokers, and can lead to a false-positive diagnosis of CF. A repeated measurement of a pathologic NPD response reduced the false-positive results in 1 out of 4 healthy controls in our cohort and should be recommended as a standard approach.

ICM was developed as a research tool for CFTR function in the 1990s [31] and has been used as a diagnostic test since the early 2000s [15, 32]. Two different protocols are established; the Freiburg protocol [9] and the original [33] and adapted [34] Rotterdam protocol. We used the newest ECFS-ICM-SOP, which is an adapted Rotterdam protocol. The combination of cAMP-mediated Cl^-^ secretion, and the carbachol and histamine (Rotterdam protocol) responses separate patients with CF from those without CF [11, 12, 34], but not patients with CF who are PS from those who are PI [11, 12], which is in accordance with our results. The 50% loss of CFTR protein in CF heterozygotes could not be detected by ICM [35] independent of the protocol [15, 32]. For the Rotterdam protocol, De Jonge postulated that the ICM response is not proportional to the CFTR amount in the apical membrane of coloncytes except at a low level (<10–15%) and could therefore only detect an 80–85% loss of CFTR expression/function [32]. Therefore, mild mutations could result in a false-negative ICM. Interestingly, Derichs reported 8 patients with a sweat chloride >60 mmol/l, fewer than two CF-causing mutations after sequencing, and a normal ICM response who were judged as CF unlikely [12]. Our results with the new ECFS-ICM SOP confirm the high predictive value and practicability of this adapted ICM Rotterdam protocol.

Our data suggest that NPD is more likely to detect CFTR dysfunction in healthy controls than the ICM or sweat test. This could be explained by tissue specific differences in *CFTR* expression, alternative chloride channel expression, or extrinsic factors. Kälin et al. showed identical *CFTR* expression in the respiratory and intestinal tract of F508del-homozygous patients and healthy controls [36]. Highly variable CFTR expression in the nose [37] and colon [38] of F508del homozygous patients has been described, varying from 0–100% [37]. Therefore, in the respiratory and intestinal tract, individual *CFTR* expression seems to be more relevant than tissue specific expression. Alternative chloride channels could contribute to the chloride conductance, but have not been described in the distal colon [39]. Furthermore, previous infections [40], milder trauma [14], smoking [17], increased paracellular permeability [41], and decreased CFTR expression [42] and CFTR response [43]. With the exception of smoking, these extrinsic factors are relevant only for NPD, but not for rectal biopsies [44]. Smoking causes a decreased NPD response [17], but although a decreased systemic CFTR function mediated by acrolein [18]. Raju et al. demonstrated a 65% decrease in the ICM CFTR response in healthy smokers compared with non-smokers [18]. Our results confirm these findings. In contrast to NPD, smoking did not influence the diagnostic cut-off for ICM in our cohort. Therefore, ICM seems to be a more robust diagnostic test than NPD to distinguish primary from secondary *CFTR* dysfunction. This is important for the interpretation of NPD as an adjunctive diagnostic test in patients with query-CF who are exposed to smoking.

Limitations of our study include the small number of participants in each group, the lack of patients with CF with a sweat chloride value < 60 mmol/l, and patients with congenital bilateral absence of the vas deferens (CBAVD). The strength of our study is the genotyping of healthy controls with an abnormal CFTR NPD response, and the use of standardized protocols for sweat testing, NPD, and ICM.

## Conclusions

From our results, a normal average NPD *CFTR* response excludes CF, but an intermediate or abnormal NPD *CFTR* response could be detected in healthy controls. NPD should be judged carefully, especially in patients with chronic rhinosinusitis and exposure to smoking. ICM combined with cAMP-mediated and cholinergic Cl secretion seems to be a practicable diagnostic test with an increased specificity compared with NPD. Discordant results of both *CFTR* functional tests could be detected and challenge the diagnostic interpretation. Larger study groups that include smokers and patients with CBAVD or CF with a sweat chloride between 30–60 mmol/l are needed to confirm our results.

## Electronic supplementary material

Additional file 1: **Correlation of the best and average Δ0Cl** ^**-**^ **+ Iso (NPD).** The normal range is shown below and left of the dotted lines. The intermediate range is shown between the solid and dotted lines. (ZIP 26 KB)

Additional file 2: **Correlation of the best and average ΔIsc (forskolin/IBMX + carbachol + histamine) (ICM).** A higher ΔIsc (forskolin/IBMX + carbachol + histamine) represents a better CFTR response. (ZIP 46 KB)
